# Investigation of the Jasmonate ZIM-Domain Family Reveals *PavJAZ8* Regulates Fruit Aroma Traits in Sweet Cherry (*Prunus avium* L.)

**DOI:** 10.3390/biom15121721

**Published:** 2025-12-11

**Authors:** Wei Wang, Tianle Shi, Zhengrong Dai, Xiaoming Zhang, Jing Wang, Chuanbao Wu, Chen Feng, Guohua Yan, Kaichun Zhang, Yuan Yang, Xuwei Duan

**Affiliations:** 1 Institute of Forestry and Pomology, Beijing Academy of Agriculture and Forestry Sciences, Beijing 100093, China; wangw@baafs.net.cn (W.W.); shitianle@baafs.net.cn (T.S.); zhxmmail@126.com (X.Z.); sduwj@126.com (J.W.); cb19902010@163.com (C.W.); fengchen@baafs.net.cn (C.F.); bigjohn6524@hotmail.com (G.Y.); kaichunzhang@126.com (K.Z.); 2 College of Agriculture and Biotechnology, Zhejiang University, Zijingang Campus, Hangzhou 310058, China; 0622520@zju.edu.cn

**Keywords:** sweet cherry, JAZ, fruit, jasmonic acid, aroma

## Abstract

Jasmonate ZIM-domain (JAZ) family genes, which belong to TIFY family, are plant-specific transcriptional repressors. As key regulators of the jasmonic acid signaling pathway, JAZ proteins play crucial roles in various aspects of plant biology. However, the identification and functional characterization of *JAZ* genes in sweet cherry (*Prunus avium* L.) fruit remain unknown. In the present study, we identified nine *JAZ* members in the sweet cherry genome. We systematically analyzed the gene structures, protein domains, evolutionary relationships, and physicochemical properties of these members and also evaluated their expression levels across different fruit developmental stages, as well as under methyl jasmonate (MeJA) treatment. Among these members, our results revealed a previously uncharacterized *JAZ* member, *PavJAZ8*, as a crucial regulator of fruit aroma traits. Specifically, RT-qPCR analysis showed that *PavJAZ8* overexpression modulates the expression of genes involved in the biosynthesis of aroma volatiles, such as *PavLOX2*, *PavLOX3*, *PavHPL1*, *PavADH1.1*, *PavADH1.2*, and *PavADH7*, which are involved in the synthesis of aldehydes and alcohols. Consistent with the gene expression data, analysis of volatile metabolites revealed that *PavJAZ8* overexpression significantly inhibited the accumulation of several related aldehydes and alcohols, including hexanal, geraniol, and benzyl alcohol. Furthermore, *PavJAZ8* expression was highly responsive to phytohormone treatments, such as abscisic acid (ABA) and MeJA. Further analysis showed that *PavJAZ8* interacts with *PavMYC2*, thereby mediating JA signal transduction. Our results highlight *PavJAZ8* as a novel regulator of fruit aroma quality, offering a valuable genetic target for sweet cherry improvement.

## 1. Introduction

Sweet cherry (*Prunus avium* L.) is an important fruit worldwide due to its favorable flavor, rich nutrition and high commercial interest [1,2]. As non-climacteric fruits, sweet cherries exhibit complex regulatory mechanisms governing ripening and quality formation [3,4], which are jointly regulated by various phytohormones, including abscisic acid (ABA), auxin (IAA), and gibberellins (GAs) [5,6,7,8,9]. In recent years, increasing evidence suggests that jasmonates (JAs) also play important roles in the ripening and quality formation of non-climacteric fruits [10,11,12,13,14]. This is supported by observations that jasmonic acid (JA) content increases during fruit ripening [15] and that exogenous treatment with JAs can significantly accelerate fruit ripening and flavor formation [10,13]. Consistently, a number of studies have demonstrated that the application of JAs promotes the accumulation of a variety of aroma compounds [16] and quality-associated compounds, such as anthocyanins [17,18]. Furthermore, JA-mediated crosstalk with other hormones, including salicylic acid (SA) and ethylene, modulates defense responses that are intrinsically linked to fruit quality [19].

The role of JA in fruit ripening and flavor formation is further exemplified in sweet cherry, with JA content in the exocarp increasing during early fruit development and remaining stable until harvest, suggesting a role for JA in promoting ripening [20]. Kondo et al. (2002) demonstrated that JA concentration may influence ABA levels, and endogenous JA could also regulate cell division during fruit development [21]. Additionally, a recent study showed that exogenous methyl jasmonate (MeJA) application at the fruit set stage alters the wax composition of ripe sweet cherry cuticles, particularly by enhancing alkane biosynthesis [22]. As alkanes are major components of the cuticular wax, this enhancement likely fortifies the cuticle—a key barrier crucial for limiting water loss, defending against pathogens, and maintaining fruit firmness and appearance, thereby directly contributing to improved fruit quality.

The JA signaling cascade comprises several critical components: the F-box subunit of the SCF-COI1 ubiquitin ligase, known as CORONATINE-INSENSITIVE1 (COI1) [23]; the JASMONATE-ZIM DOMAIN (JAZ) protein repressors [24,25,26]; and the basic helix-loop-helix transcription factors (MYC) [27]. The bioactive jasmonate ligand, (3R,7S)-jasmonoyl-L-isoleucine (JA-Ile) [28], binds to COI1, triggering JAZ protein degradation via the ubiquitin-proteasome pathway. This releases MYC2 and other transcription factors from repression, leading to the activation of JA-responsive genes [25,26,27,29].

JAZ family proteins are plant-specific transcriptional repressors belonging to the TIFY family. Studies have identified 13 *JAZ* genes in Arabidopsis (*Arabidopsis thaliana*) [24], 16 in maize (*Zea mays*) [30], and 26 in tomato (*Solanum lycopersicum*) [31]. As core components of the JA signaling cascade, JAZ proteins have been increasingly studied for their roles in fruit ripening and quality formation. A study in strawberry (*Fragaria vesca*) reported that expression levels exhibited a significant decrease from fruit development to ripening stages, suggesting that *FveJAZs* may be important factors regulating fruit ripening [32]. Similarly, in strawberry fruits, Wang et al. [33] found that *FveJAZ12* can mediate the transduction of JA signaling through a COI1-independent pathway, regulating fruit quality traits, particularly aroma compound metabolism. In kiwifruit (*Actinidia chinensis*), *AchJAZ9* expression increased gradually during fruit ripening, while *AchJAZ3* and *AchJAZ11* were highly expressed in the ripening stage. The diversity of expression patterns during fruit ripening suggests that these *AchJAZs* may play different roles [34]. In apple (*Malus domestica*), Hu Y et al. [35] showed that *MdoJAZ* is a core JA signaling hub protein that affects ethylene signaling pathways, and suppressing *MdoJAZ* expression promotes apple ripening and fruit trait development.

Although the *JAZ* gene family has been identified in various horticultural crops [31,32,36,37,38,39], a systematic characterization in sweet cherry is lacking. To address this gap, we identified nine *PavJAZ* family members in sweet cherry, and subsequently analyzed their protein structure, conserved domains, chromosome locations, and other features. We also investigated their stage-specific expression patterns and responses to MeJA. Among them, we uncovered a previously uncharacterized *JAZ* gene, *PavJAZ8*, which is highly expressed throughout all fruit stages. Its transcription can be significantly modulated by both MeJA and ABA treatments. *PavJAZ8* physically interacts with *PavMYC2*. Furthermore, functional evidence indicates that *PavJAZ8* regulates fruit aroma quality. Transient overexpression of *PavJAZ8* altered the levels of key green and fruity volatiles, such as (E)-2-hexenal and geraniol. Consistent with this phenotypic change, it modulated the expression of critical biosynthetic genes, including *PavHPL1*, *PavLOXs*, and *PavADHs*. These results provide a basis for further research on the role of JAZ proteins in sweet cherry fruit ripening and trait development.

## 2. Materials and Methods

### 2.1. Plant Materials and Phytohormone Treatments

Sweet cherry (*Prunus avium* L.) cv ‘Tieton’ was grown in the Beijing Academy of Agriculture and Forestry Sciences, Beijing, China. For *PavJAZs* gene expression analysis, fruit samples were classified into six developmental stages, including the Small Green (SG, 1–2 weeks after flowering), Middle Green (MG, 3–4 weeks after flowering), Large Green (LG, 5 weeks after flowering), Turning (T, 6 weeks after flowering), Initial Red (IR, 7–8 weeks after flowering), and Full Red (FR, 9 weeks after flowering) stages.

Phytohormone treatments were applied to fruits at the LG stage. Fruits with uniform sizes and normal shape were harvested and treated by vacuum infiltration with 100 μM MeJA, 100 μM ABA, 100 μM GA_3_, 100 μM SA, with distilled water as the control. At predetermined time points, fruits were snap frozen in liquid nitrogen and stored at −80 °C for subsequent molecular analyses. The experiment included three biological replicates, with 10 fruits per treatment in each replicate.

### 2.2. Identification and Classification of JAZ Gene Family in Sweet Cherry

The *JAZ* gene family of Arabidopsis was retrieved from the TAIR database (https://www.arabidopsis.org/). These accession numbers are provided in Table S1. Using the Arabidopsis JAZ protein sequences as queries, homologous *JAZ* family genes were identified in sweet cherry. The *Prunus avium* Tieton Genome v2.0 [40] sequences were downloaded from the Genome Database for Rosaceae (https://www.rosaceae.org/). Accession numbers for each of the identified *PavJAZs* are listed in Table S1. Physicochemical properties of *PavJAZs*, including theoretical isoelectric point and molecular weight, were predicted using the ExPASy Proteomics Server (https://web.expasy.org).

### 2.3. Chromosomal Location and Phylogenetic Analysis of PavJAZs

Chromosomal localization of *PavJAZs* was obtained based on *Prunus avium* cv. Tieton v2.0 genome annotation information. Subsequently, TBtools v0.66836 [41] was used to map and visualize chromosome distributions. Intra- and inter-species *PavJAZs* collinearity analyses were performed using TBTools. Non-synonymous substitution rate (*Ka*) and synonymous substitution rate (*Ks*) were calculated using *KaKs*_Calculator 2.0 [42].

Using the Arabidopsis JAZ protein sequences as queries, homologous *JAZ* family genes were identified in sweet cherry (*Prunus avium* Tieton Genome v2.0), apricot (*Prunus armeniaca* Yinxiangbai v1.0) [43], peach (*Prunus persica* Zhongyoutao 14 Genome v1.0) [44], strawberry (*Fragaria vesca* Genome v4.0.a2) [45], and tomato (SL4.0 build with ITAG4.0 annotation) [46] using BLAST v2.2.28+. The presence of the conserved domain in candidate proteins was verified with the NCBI Batch Web CD-Search Tool (https://www.ncbi.nlm.nih.gov/Structure/bwrpsb/bwrpsb.cgi, accessed on 20 May 2025) with default parameters. Subsequently, a phylogenetic analysis was conducted using the JAZ protein sequences from these six species. A multiple sequence alignment was generated using MUSCLE v3.8.425 with default settings, and low-quality regions were filtered using trimAl v1.4.rev15. The trimmed alignments were used to construct a maximum likelihood phylogenetic tree with IQ-TREE v1.6.7. The best-fitting model determined by ModelFinder was Q.plant + I+G4. Branch support was assessed with 1000 bootstrap replicates.

### 2.4. Structural and Domain Analysis of PavJAZs

Protein sequence alignments of *PavJAZs* were performed using Clustal X 2.1 [47] and visualized with Jalview [48]. Gene structure diagrams were generated using Gene Structure Display Server (GSDS) 2.0 (http://gsds.gao-lab.org/). Protein domains were identified using the Pfam database (http://pfam.xfam.org/), and sequence logos of the TIFY and JAS domains were generated using WebLogo (http://weblogo.berkeley.edu/logo.cgi, accessed on 20 May 2025).

### 2.5. RNA Extraction and Quantitative Real-Time PCR (RT-qPCR) Analysis

RNA extraction and RT-qPCR were performed as previously described [49]. Briefly, total RNA was extracted from fruit samples using the Plant RNA Kit (Omega, Norcross, GA, USA, R6827-01). First-strand cDNA was synthesized using the HiScript II 1st Strand cDNA Synthesis Kit (+gDNA wiper) (Vazyme, Nanjing, China, R212-01) following the manufacturer’s protocol. RT-qPCR was performed on a QuantStudio 6 Flex Real-time PCR system using ChamQ SYBR qPCR Master Mix (Vazyme, Nanjing, China, Q311-02). The RT-qPCR data were analyzed using the 2^−ΔΔCt^ method for calculating relative gene expression, with *PavACTIN* serving as the internal reference gene. Gene-specific primers are listed in Table S2.

### 2.6. Gas Chromatography-Mass Spectrometry Analysis of Volatile Metabolites

Sweet cherry fruit samples were immediately frozen using liquid nitrogen and subsequently subjected to gas chromatography-mass spectrometry (GC-MS). VOC desorption from the SPME Arrow fiber was performed in an GC injector at 250 °C for 5 min. Separation and detection of volatile compounds were conducted using an Agilent 8890 (Agilent Technologies, Santa Clara, CA, USA) GC system coupled with a 7000E mass spectrometer. A DB-5MS capillary column was employed for chromatographic separation, with helium as the carrier gas maintained at a constant flow rate of 1.2 mL/min. The oven temperature program started at 40 °C, followed by a ramp of 10 °C/min to 100 °C, then 7 °C/min to 180 °C, and finally 25 °C/min to 280 °C, with a final hold time of 5 min. MS detection was performed in electron ionization mode at 70 eV. The temperatures of the quadrupole, ion source, and transfer line were set to 150 °C, 230 °C, and 280 °C, respectively. Analytes were identified and quantified using selected ion monitoring mode.

### 2.7. Transient Fruit Transformation

Transient transformation assays in sweet cherry fruits were performed using *Agrobacterium tumefaciens*-mediated infiltration, following an established protocol [50] with modifications. Fruits at the LG developmental stage were selected for transformation. The coding sequences of *PavJAZ8* were cloned and inserted into the pSuper1300-GFP vector. The GV3101 *Agrobacterium* strains carrying pSuper1300-*PavJAZ8*-GFP or pSuper1300-GFP vectors were cultivated to an optical density (OD) of 0.8 at 600 nm (OD_600_), followed by infiltration into the fruits.

### 2.8. Subcellular Localization Analysis

The subcellular localization of the *PavJAZs* was predicted by the online web-servers tool Cell-PLoc 2.0 [51] and WoLF PSORT (https://wolfpsort.hgc.jp/). For experimental validation, the coding sequence of *PavJAZ8* was cloned into the pSuper1300-GFP vector to generate a C-terminal green fluorescent protein (GFP) fusion. The recombinant *Agrobacterium tumefaciens* strain GV3101 was cultivated to an OD_600_ of 0.6–0.8, and then infiltrated into leaves of transgenic *N. benthamiana* plants expressing a nuclear-localized mCherry marker to achieve transient transformation [52]. After 2–3 days, subcellular localization was analyzed with a Zeiss LSM 710 confocal microscope. Samples were excited with a 488 nm argon laser. The emitted fluorescence was collected within a spectral window of 500–550 nm.

### 2.9. Bimolecular Fluorescence Complementation

To investigate protein–protein interaction between *PavJAZ8* and *PavMYC2*, the bimolecular fluorescence complementation (BiFC) assays were performed using the pSPYNE-35S and pSPYCE-35S vectors [53]. The vectors were transiently expressed in *N. benthamiana* leaves via agroinfiltration using *Agrobacterium tumefaciens* strain GV3101 [52]. After 2–3 days, fluorescent signal was visualized with a Zeiss LSM 710 confocal microscope. Samples were excited with a 488 nm argon laser line. The emitted fluorescence was collected within a spectral window of 500–550 nm.

### 2.10. GST Pull-Down Assay

A GST pull-down assay was performed to test for a direct physical interaction between *PavJAZ8* and *PavMYC2*.

Protein Expression and purification: The coding sequences of *PavJAZ8* and *PavMYC2* were cloned into the pGEX-4T-2 and pET-30a vectors, respectively. The recombinant plasmids were transformed into *E. coli* strain BL21 (DE3). For protein expression, cultures were grown at 37 °C to an OD_600_ of 0.6–0.8. Expression was induced by adding 1 mM isopropyl β-d-1-thiogalactopyranoside, followed by incubation at 16 °C for 12 h. Cells were harvested by centrifugation and lysed by sonication on ice. The HIS-tagged *PavMYC2* protein was purified from the soluble fraction using Ni-NTA agarose beads. The beads were incubated with the lysate for 1 h at 4 °C, then washed extensively with a wash buffer (50 mM Tris-HCl, pH 8.0, 300 mM NaCl, 20 mM imidazole). Bound proteins were eluted with an elution buffer containing 250 mM imidazole. The GST-tagged *PavJAZ8* was purified under similar conditions using Glutathione Sepharose 4B beads.

Pull-down Assay: For the pull-down assay, purified *PavJAZ8*-GST (experimental group) or GST alone (negative control) was immobilized on Glutathione Sepharose 4B beads. The beads were then incubated with an equal amount of purified *PavMYC2*-HIS protein for 2 h at 4 °C with gentle rotation. After incubation, the beads were washed three times with ice-cold PBS buffer to remove non-specifically bound proteins. Protein interactions were analyzed by immunoblotting using Anti-GST (Abclonal, Wuhan, China, AE001, 1:5000) and Anti-HIS (Abclonal, Wuhan, China, AE003, 1:5000) antibodies to detect the bait (*PavJAZ8*-GST) and prey (*PavMYC2*-HIS) proteins, respectively.

## 3. Results

### 3.1. Identification and Classification of the PavJAZ Genes

Nine putative *PavJAZs* were identified by blast (BLAST v2.2.28+) with *JAZ* gene sequences in Arabidopsis and further validated for the presence of structural domains using the NCBI Batch Web CD-search tool with default parameters. These members were classified and named according to the chromosomal locations of the genes.

Physicochemical analysis showed that the encoded *PavJAZ* family members ranged from 124 to 377 amino acids (aa), with coding sequence (CDS) lengths of 375–1134 base pairs (bp). The theoretical molecular weights (MW) of these proteins were calculated to be in the range of 14.66–40.40 kDa, and the predicted isoelectric points (pI) ranged from 6.18 to 9.80. Notably, most *PavJAZ* proteins exhibited an isoelectric point above 8.5, except *PavJAZ8*, which had an isoelectric point of 6.18. The grand average of hydropathicity (GRAVY) index for all *PavJAZs* was negative, indicating their hydrophilic nature. Additionally, all proteins were predicted as unstable, with instability index values exceeding 40 (Table 1). This inherent instability is consistent with their canonical role as repressors in the jasmonate signaling pathway, which are rapidly degraded by the proteasome following hormone perception. This finding is also in agreement with the unstable nature of JAZ proteins reported in other plant species [25,26,29], further supporting that rapid turnover is a conserved characteristic of this protein family.

### 3.2. Chromosomal Locations and Collinearity Analysis of PavJAZs

**Figure 1:**
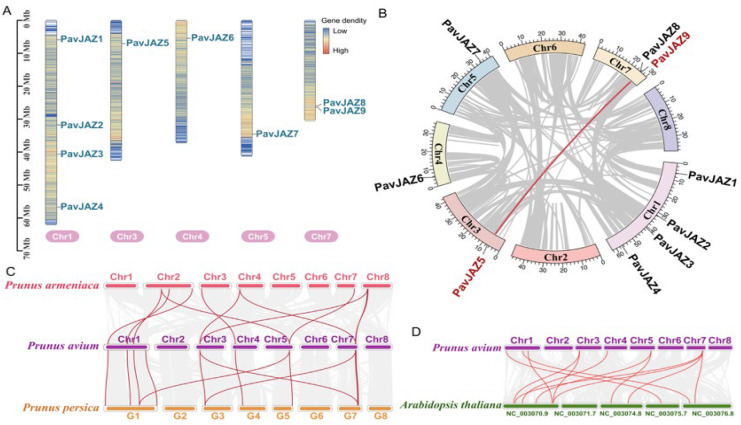
Chromosomal distribution and collinearity analysis of *PavJAZs*. (**A**) Chromosomal distribution of *PavJAZ* genes in sweet cherry. (**B**) Intra-genomic synteny of *PavJAZ* genes in sweet cherry. The only duplicated pair (*PavJAZ5/PavJAZ9*, connected by red line) suggests a segmental duplication event. (**C**,**D**) Collinearity analysis of *JAZ* genes in *Prunus* species (sweet cherry, peach, and apricot) and syntenic relationships between sweet cherry and Arabidopsis *JAZ* genes. Red lines indicate gene pairs that are collinear with *JAZ* genes.

The *PavJAZ* genes were unevenly distributed across 5 out of the 8 sweet cherry chromosomes. As shown in Figure 1A, Chromosomes 1 and 7 harbor 4 and 2 *PavJAZ* genes, respectively. Chromosomes 3, 4, and 5 each contain 1 *PavJAZ* gene. No *PavJAZ* genes were mapped to chromosomes 2, 6, or 8.

To investigate *PavJAZ* genes duplication events within the sweet cherry genome, we performed whole-genome collinearity analysis and identified only one duplicated *PavJAZ* pair (*PavJAZ5* and *PavJAZ9*) (Figure 1B), suggesting that these two *JAZ* genes are derived from a common ancestral gene via segmental duplication. Evolutionary selection pressure analysis revealed that the *Ka*/*Ks* ratio values < 1 for this gene pair (Table 2), indicating strong purifying selection acting on this gene pair. To further investigate conserved syntenic relationships across *Prunus* species, we conducted whole-genome synteny analysis between sweet cherry, peach, and apricot. Genomic synteny analysis showed a high degree of conservation between sweet cherry and its close relatives, peach and apricot (Figure 1C), indicating strong chromosomal stability within the *Prunus* genus (Rosaceae). Notably, compared to peach, sweet cherry and apricot exhibited more chromosomal rearrangements, and some *JAZ* genes were located within these rearranged genomic regions. In addition, synteny analysis between sweet cherry and Arabidopsis revealed extensive genomic divergence, while the *JAZ* gene structure remained relatively conserved (Figure 1D).

### 3.3. Phylogenetic Analysis of JAZs

**Figure 2:**
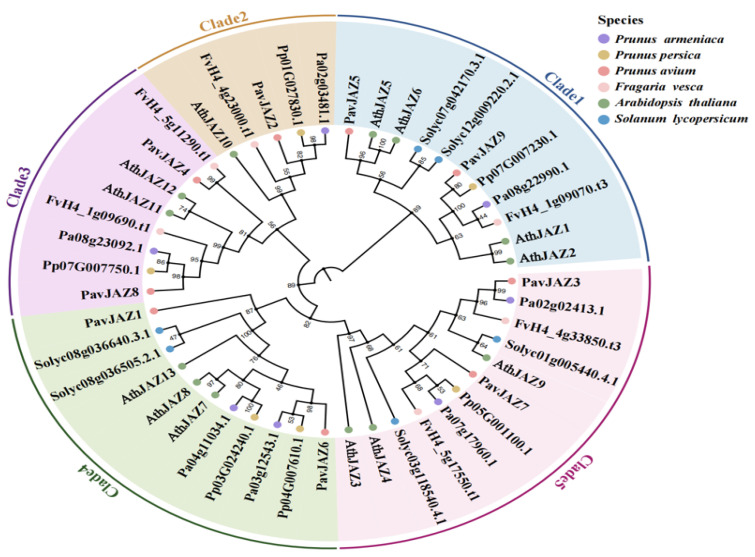
Phylogenetic analysis of JAZ proteins in six species. The phylogenetic tree was constructed using 47 JAZ protein sequences from *Arabidopsis*, strawberry, tomato, apricot, peach, and sweet cherry.

To elucidate the evolutionary relationship of *JAZ* genes among plant species, we analyzed 47 JAZ protein sequences from six species, including nine from sweet cherry, 13 from Arabidopsis, six from tomato, six from strawberry, six from peach, and seven from apricot (Figure 2). The number of *JAZ* genes in sweet cherry did not differ significantly from those in peach and apricot, but there was a significant contraction compared with Arabidopsis. Maximum likelihood phylogenetic trees showed that *PavJAZs* were closely clustered with the immediate homologues of related species (Figure 2), suggesting that *JAZ* genes are highly evolutionarily conserved.

### 3.4. Protein Structure, Conserved Domains Analysis of PavJAZs

**Figure 3:**
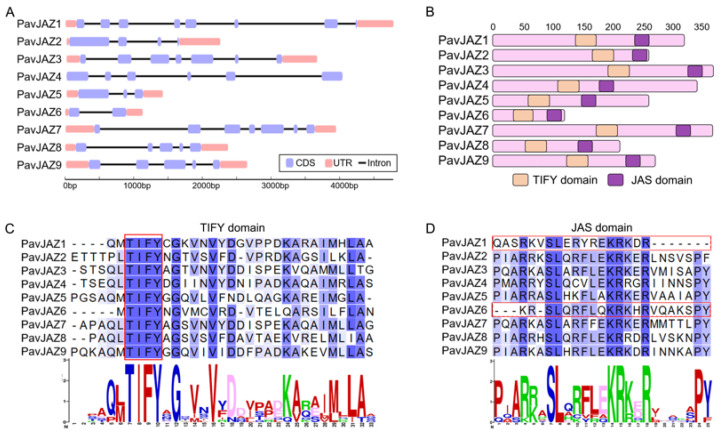
Gene and protein structures including conserved domain analysis of *PavJAZs*. (**A**) Exon-intron structure of *PavJAZ* genes. (**B**) *PavJAZ* proteins contain the conserved TIFY and JAS domains. (**C**) Sequence alignment of the TIFY domains in *PavJAZ* proteins. (**D**) Sequence alignment of the JAS domains in *PavJAZ* proteins.

Gene structure analysis showed that *PavJAZs* exhibit differences in exon-intron architecture, with the number of exons ranging from 3 to 8 (Figure 3A). Our results revealed strong conservation across *PavJAZs* domains (Figure 3B): the TIFY motif within the TIFY domain, which mediates interactions with the NINJA adapter and facilitates JAZ homo- and hetero-dimerization, was completely identical among all members (Figure 3C), while most of the *PavJAZs* contained a conserved JAS domain exhibiting the SLX_2_FX_2_KRX_2_RX_5_PY motif, which is critical for JA signal transduction (Figure 3D).

### 3.5. Expression Analysis of the PavJAZs in Different Fruit Stages

**Figure 4:**
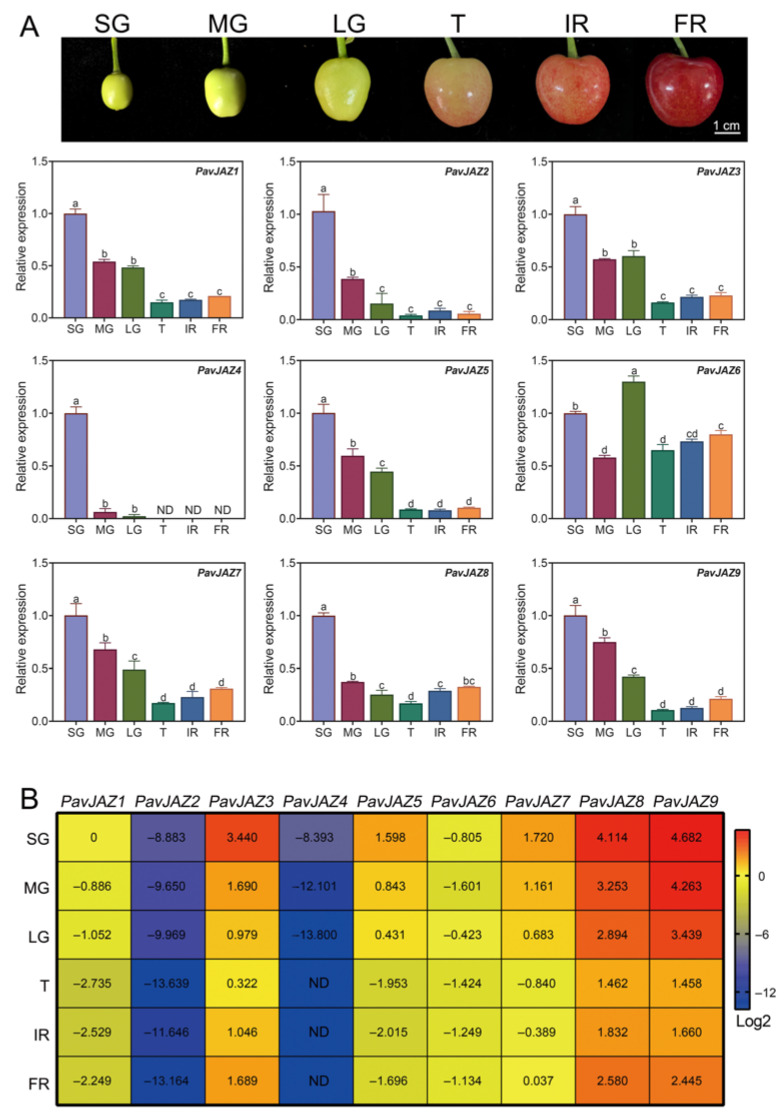
Relative expression analysis of *PavJAZs* in different fruit stages. (**A**) RT-qPCR analysis of *PavJAZ* genes expression levels in different fruit stages. Values represent the mean ± SD from three independent biological replicates. The comparative fruit phenotypes at distinct developmental stages are displayed in the images above. Fruit were classified into six developmental stages: Small Green (SG), Middle Green (MG), Large Green (LG), Turning (T), Initial Red (IR), and Full Red (FR) stages. Different letters indicate significant differences at *p* < 0.05. (**B**) The heatmap shows relative expression levels of *PavJAZ* genes, all expression data were first normalized to the housekeeping gene *PavACTIN* (provided in Table S1) to correct for experimental variations and then calculated as fold changes relative to the expression level of *PavJAZ1* at the SG stage. “ND” denotes undetectable expression levels.

To investigate the expression patterns of *PavJAZ* genes, we analyzed their expression patterns in different fruit stages. As shown in Figure 4A, *PavJAZ1, PavJAZ2, PavJAZ3, PavJAZ7, PavJAZ8,* and *PavJAZ9* exhibited similar expression trends, with downregulation expression prior to the T stage, followed by a gradual increase in their level of expression during ripening. The expression pattern of *PavJAZ6* differed across all stages. Notably, *PavJAZ8*, and *PavJAZ9* displayed higher expression levels compared to other *PavJAZ* members in every developmental stage, and their expression profiles showed more dynamic changes during fruit ripening (Figure 4B). In contrast, *PavJAZ2* and *PavJAZ4* showed low expression levels in all stages, indicating their limited functional relevance to ripening. These differential expression patterns suggest distinct roles of *PavJAZ* members in fruit ripening processes.

### 3.6. Relative Expression Levels of PavJAZs in Fruits Under MeJA Treatment

**Figure 5:**
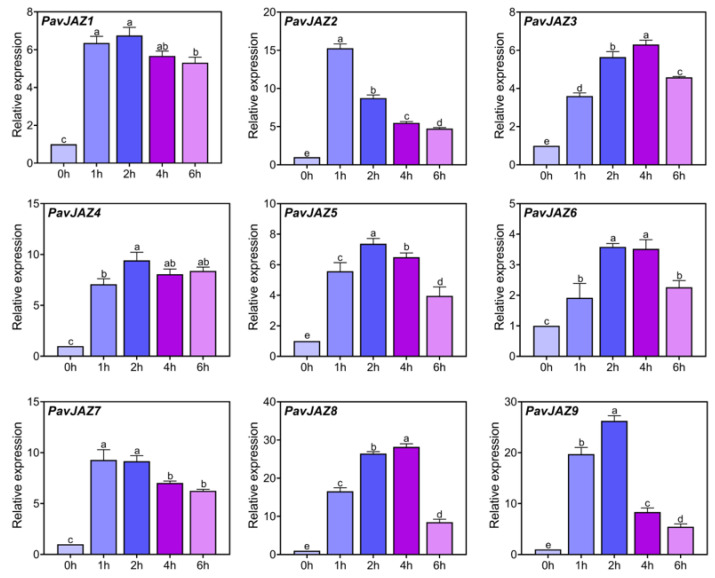
RT-qPCR analysis of *PavJAZ* genes expression levels in MeJA-treated fruits. Values represent the mean ± SD from three independent biological replicates. Gene expression levels are shown relative to the 0 h time point. Different letters indicate significant differences at *p* < 0.05.

As core components of JA signaling, JAZ proteins mediate JA responses through transcriptional and post-transcriptional regulation. To examine the responsiveness of *PavJAZ* genes to JA, developing fruits at the LG stage were treated with 100 μM MeJA. As shown in Figure 5, all *PavJAZ* genes displayed significant upregulation within one hour after MeJA treatment, reaching peak expression levels at 2 to 4 h post treatment. Notably, *PavJAZ8* and *PavJAZ9* exhibited the most pronounced and sustained induction, with the highest fold-change across multiple time points. Although their expression declined at 6 h, *PavJAZ8* levels remained more than 8.46-fold higher than the baseline (0-h), while *PavJAZ9* levels were 5.44-fold higher.

### 3.7. PavJAZ8 Regulates Sweet Cherry Fruit Aroma Traits

Aroma quality is a core component of the commercial value of sweet cherries. This aroma profile is derived from approximately 50 volatile compounds. These compounds range from the green, grassy aldehydes characteristic of unripe fruit to the fruity, floral esters and alcohols of ripe fruit [54,55]. This shift in volatile balance is therefore critical for aroma development during ripening, a process in which jasmonates have been shown to play a significant role [55].

**Figure 6:**
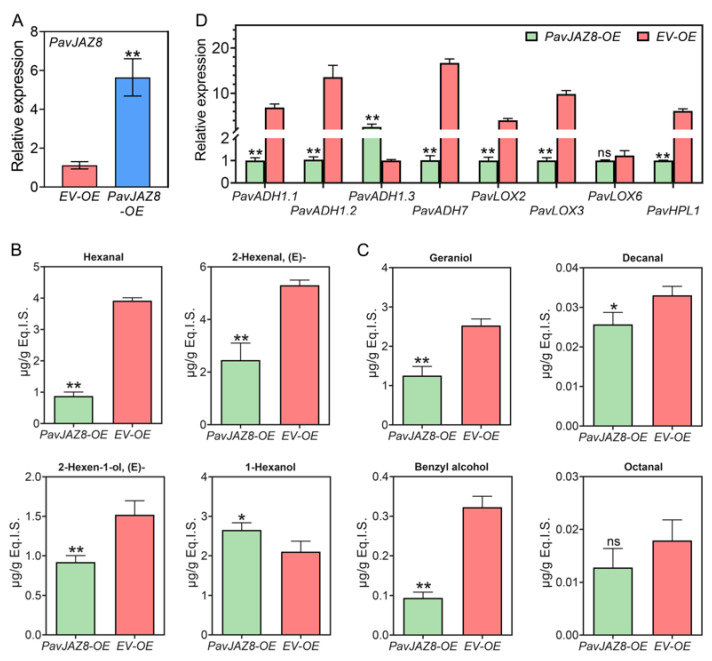
*PavJAZ8* overexpression regulates aroma compound biosynthesis in early stage sweet cherry fruits. (**A**) Transient overexpression of *PavJAZ8* was achieved in the fruit. (**B**) The contents of key green compounds differed between *PavJAZ8-OE* and *EV-OE*. (**C**) The contents of key fruity compounds differed between *PavJAZ8-OE* and *EV-OE*. (**D**) Expression levels of aroma biosynthesis-related genes in *PavJAZ8-OE* and *EV-OE*. Values represent the mean ± SD from three independent biological replicates. Asterisks denote statistically significant differences (* *p* < 0.05, ** *p* < 0.01, ns, not significant). The corresponding gene IDs are provided in Table S1.

During early fruit development, *PavJAZ8* was the second most highly expressed *PavJAZ* gene, behind only *PavJAZ9*. However, its expression became the highest during the later ripening stages (T, IR, and FR). This high-level expression, together with its strong induction by MeJA, suggests a key role in the MeJA-mediated regulation of trait development. To further elucidate the function of *PavJAZ8* during fruit aroma biosynthesis, we transiently overexpressed *PavJAZ8* in LG stage fruits via *Agrobacterium*-mediated infiltration. Seven days after inoculation, the expression level of *PavJAZ8* in OE (*PavJAZ8-OE*) fruits was more than fivefold higher than that in the empty vector (*EV-OE*) fruits, a difference that was statistically significant (Figure 6A).

We analyzed the contents of eight main green and fruity compounds in sweet cherry from *PavJAZ8-OE* and *EV-OE* using GC-MS. As shown in Figure 6B, among the four key green compounds detected, the level of (E)-2-hexenal, was reduced to less than half in *PavJAZ8-OE* compared to *EV-OE*, while the levels of hexanal and (E)-2-hexen-1-ol were also significantly decreased in *PavJAZ8-OE*, to approximately 22.4% and 60.6% of the *EV-OE* levels, respectively. In contrast, the content of 1-hexanol showed an upward trend in *PavJAZ8-OE*. These results suggest that *PavJAZ8* may suppress the emission of green fruit odors, thereby influencing flavor. Figure 6C illustrates the effect of *PavJAZ8* overexpression on the relative contents of the main fruity compounds detected in sweet cherry. The relative contents of geraniol, decanal, and benzyl alcohol were also reduced in *PavJAZ8-OE* samples compared to *EV-OE*, while no significant differences were found for octanal. This dual effect suggests that *PavJAZ8* directs a complex and multifaceted role in regulating fruit aroma profiles. To understand the molecular basis of these changes, RT-qPCR analysis of key aroma-related gene expression showed that the transient overexpression of *PavJAZ8* altered the expression of several critical biosynthetic genes, including *PavHPL1*, *PavLOX2*, *PavLOX3*, *PavADH1.1/1.2/1.3,* and *PavADH7*, which are involved in the synthesis of aldehydes and alcohols (Figure 6D). Collectively, our results demonstrate that *PavJAZ8* acts as a master regulator of aroma profiles in sweet cherry, modulating the production of both green and fruity volatile compounds.

### 3.8. Expression Patterns of PavJAZ8 After Phytohormone Treatment

Given the critical roles of phytohormones in fruit ripening and trait development, we investigated the effects of ABA, SA, and GA_3_ on *PavJAZ8* expression in sweet cherry fruits using RT-qPCR. As shown in Figure S1. *PavJAZ8* transcript levels began to increase 1 h after ABA treatment, peaked at 2 h with approximately a 10-fold upregulation, and then gradually declined. SA treatment induced a similar pattern, peaking at 2 h with a ~3-fold upregulation before returning to the baseline. In contrast, GA_3_ treatment resulted in a slightly different response, with *PavJAZ8* expression reaching its maximum level at 4 h after GA_3_ treatment and then decreasing sharply. Notably, among the three hormones tested, ABA exhibited the most pronounced effect in inducing *PavJAZ8* expression in fruits.

### 3.9. Subcellular Localization Analysis of PavJAZ8

As a transcription repressor, the JAZ protein generally functions by associating with transcription factors in the nucleus. Studies in Arabidopsis and other plant species have indicated that JAZ proteins are predominantly expressed in the nuclei, with occasional cytoplasmic localization. To determine the subcellular localization of *PavJAZ* proteins, we first analyzed nine *PavJAZ* proteins using the online prediction tools. The results revealed that all nine proteins were predicted to localize in the nucleus (Table S3).

**Figure 7:**
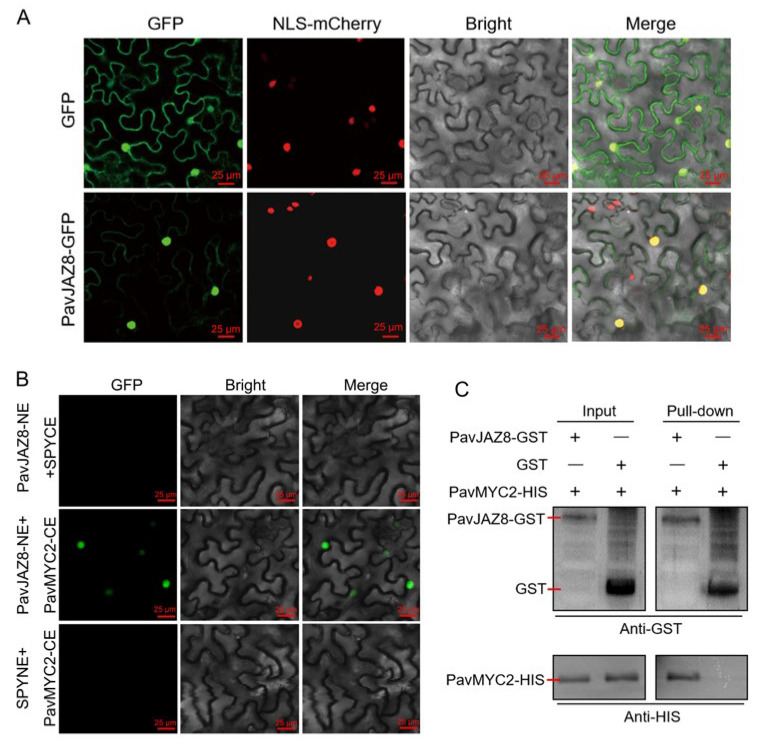
*PavJAZ8* Interacts with *PavMYC2* in the nucleus. (**A**) Subcellular localization of *PavJAZ8* was observed using confocal laser scanning microscopy, with the GFP empty vector serving as a control. Scale bar = 25 μm. (**B**) BiFC assay demonstrated that *PavJAZ8* and *PavMYC2* physically interact in the nucleus. Scale bar = 25 μm. (**C**) GST pull-down assay confirmed a direct physical interaction between *PavJAZ8* and *PavMYC2*.

To experimentally validate the subcellular localization of *PavJAZ8*, the pSuper1300-*PavJAZ8*-GFP construct and the empty vector were introduced into the leaves of *N. benthamiana*. As demonstrated in Figure 7A, the empty vector control displayed GFP expression in both the nucleus and cytoplasm, whereas the *PavJAZ8*-GFP fusion protein exhibited predominantly nucleus-localized expression, with cytoplasmic signals detected in only a minor subset of cells. These results support that *PavJAZ8* is primarily localized in the nucleus, consistent with its predicted role in transcriptional regulation.

### 3.10. PavJAZ8 Interacts with the PavMYC2

Previous studies established MYC2 as a core transcription factor directly regulated by JAZ proteins in JA signaling [27,29]. Moreover, MYC2 plays a crucial role in regulating fruit ripening and quality formation in various species, including strawberry and orange (*citrus sinensis*) [15,33]. In sweet cherry, *PavMYC2* has been identified and functionally characterized in previous studies [56]. To further elucidate the role of *PavJAZ8* in fruit JA signaling, we examined the interaction of *PavJAZ8* with *PavMYC2*. Both bimolecular fluorescence complementation (BiFC) and GST pull-down assays confirmed direct binding between *PavJAZ8* and *PavMYC2* (Figure 7B,C). These results demonstrate that *PavJAZ8* physically interacts with *PavMYC2*, an interaction that strongly suggests their likely functional involvement in the JA signaling pathway in sweet cherry.

## 4. Discussion

As key repressors of the JA signaling pathway, the JAZ proteins play crucial roles in various aspects of plant biology, including growth, development, and stress responses [33,57,58,59,60]. However, the identity and function of each *JAZ* gene in sweet cherry fruit remains unknown. In this study, we identified nine *JAZ* family members in the sweet cherry genome, which is fewer than the number reported in the model dicot species Arabidopsis (10 members), and also fewer than those in another Rosaceae species, strawberry (12 members). Similar to that in sweet cherry, the number of *JAZ* family members is also relatively low in other species of the genus *Prunus*, such as peach (6 members) and apricot (7 members). The relatively compact size of the *JAZ* family in sweet cherry and other *Prunus* species, such as peach and apricot, contrasts with the expanded family in Arabidopsis. This discrepancy may be linked to the distinct biological traits and life histories of these species. The smaller, conserved set of JAZ proteins in *Prunus* might represent a core repertoire that effectively governs the JA signaling pathways essential for their perennial tree growth habit and fruit development.

The JAZ repressors share two conserved functional domains: the TIFY (ZIM) domains in the central part of the protein and the JAS domain at the C-terminus. Studies report that the TIFY domain, which contains a conserved TIFY sequence (TI (F/Y) XG), mediates the interactions with the adapter NINJA [61] and the homo-and hetero-meric interactions among JAZ proteins [62]. The JAS domain contains two conserved sequence motifs, including the LPIAR (R/K) motif and the SLX_2_FX_2_KRX_2_RX_5_PY motif. The N-terminal LPIAR (R/K) motif functions as the COI1 binding site. This peptide sequence is termed the degron and is responsible for the stability of the COI-JA-Ile-JAZ complex [25]. The SLX_2_FX_2_KRX_2_RX_5_PY motif is essential for the subcellular localization of each JAZ protein as well as the interactions with MYC2 or other target proteins [63,64]. By analyzing the domains and motifs of the identified *PavJAZ* members, we found that sweet cherry JAZ proteins exhibit a relatively high degree of homology with those in other species (Figure 3). The motifs within the TIFY and JAS domains are highly conserved, with minimal variations. This high level of conservation demonstrates the central importance of these domains and underscores the evolutionary and functional consistency of the JA signaling machinery across species.

The spatiotemporal expression pattern of a gene often reflects its functional relevance. In our study, *PavJAZ8* exhibited consistently high expression levels in cherry fruits across all developmental stages (Figure 4B), suggesting its likely involvement throughout fruit development. Overexpression of *PavJAZ8* modulated the abundance of major contributors of sweet cherry aroma metabolites, such as Hexanal, 2-Hexenal, (E)-, Geraniol, and Benzyl alcohol. Furthermore, the overexpression of *PavJAZ8* significantly suppressed the expression of key aroma biosynthesis genes, including *PavLOXs* (aldehydes, alcohols, and esters), *PavHPL* (aldehydes), and *PavADHs* (alcohols). This directly establishes *PavJAZ8* as a component of the regulatory network for aroma quality and validates its role as a regulator in aroma biosynthesis. Therefore, we identified *PavJAZ8* as a key regulator of cherry fruit aroma traits. Notably, the identified functional role for *PavJAZ8* mirrors that of its strawberry ortholog, *FveJAZ12* (FvH4_1g09690), a known regulator of strawberry fruit trait development [33], revealing a conserved JAZ-mediated regulatory module for fruit quality traits within the Rosaceae family.

Moreover, the *PavJAZ8*-mediated regulation of specific volatiles, including aldehydes and terpenoids, implicates this regulatory module in the control of postharvest physiology, well beyond their contribution to aroma. These compounds can function as natural fumigants against pathogens and as signaling molecules that prime defense responses. Simultaneously, alterations in terpenoids like geraniol may influence the ethylene signaling network, directly impacting the rate of fruit senescence. Consequently, targeted manipulation of the JA pathway through *PavJAZ8* emerges as a sophisticated strategy to not only refine flavor profiles but also to bolster innate defense and delay senescence, ultimately improving the postharvest quality and longevity of sweet cherry.

Notably, in addition to its distinct expression pattern and specific responsiveness to JA, *PavJAZ8* is the only *PavJAZ* with an isoelectric point below 7.0. Since the isoelectric point of a protein influences its interactions with DNA or proteins, subcellular localization, and other biological functions, the unique pI of *PavJAZ8* likely contributes to functional divergence from other *PavJAZ* proteins. We speculate that the acidic nature of *PavJAZ8* could modulate its affinity for specific protein partners, such as the MYC transcription factors, or influence its degradation kinetics and other post-translational modifications. These distinctive features position *PavJAZ8* as a high-priority candidate for future investigations into the specialized regulation of JA signaling in sweet cherry.

The MYC2 transcription factor, a direct downstream target of JAZ repressors in the JA signaling pathway, has been demonstrated to regulate fruit ripening and trait development in strawberry and orange through transcriptional activation of ripening-related genes [15,33]. To investigate whether *PavMYC2* acts as a downstream component of *PavJAZ8* in sweet cherry, we employed BiFC and pull-down assays, which confirmed their physical interaction. These findings not only substantiate *PavJAZ8* as a functional repressor within the JA pathway but also offer deeper mechanistic insight into its role in modulating key fruit traits. By physically interacting with *PavMYC2*, *PavJAZ8* likely tunes the expression of ripening-related genes downstream of *PavMYC2*, thereby integrating jasmonate signaling with fruit developmental cues.

Recent studies have demonstrated that JA engages in extensive crosstalk with hormones including SA, ethylene, GAs, and ABA via JAZ proteins, thereby regulating diverse processes from development to environmental stress and defense responses [19,35,57,60,65,66,67]. This raises a question: can the process of *PavJAZ8* regulating fruit quality traits also be co-regulated by multiple hormones? Our results provide key evidence to support this hypothesis: beyond JAs, *PavJAZ8* expression is significantly regulated by ABA and GA_3_ (Figure S1). This finding carries clear functional implications, as ABA and GA_3_ play well-documented, often antagonistic roles in sweet cherry fruit ripening and quality traits: ABA accelerates pericarp coloration, sugar accumulation, and softening [68,69], while GAs delay these processes by suppressing ripening-related gene expression [70,71]. The regulation of *PavJAZ8* by both hormones suggests that it may act as a hormonal integrator, thereby integrating ABA and GA signals into the JA pathway to avoid premature or delayed ripening. This regulatory mechanism is consistent with the broader role of JAZ proteins in hormone crosstalk, positioning *PavJAZ8* as a key mediator of the multi-hormone network governing cherry fruit ripening and aroma traits. However, it is important to acknowledge the limitations of our study. Given that the transient fruit transformation system is associated with physical damage and inherent instability, future studies will be based on a stable genetic transformation system to systematically analyze the biological functions of *PavJAZ8* and its regulatory roles in fruit ripening and trait development.

## 5. Conclusions

In this study, nine *PavJAZ* genes were identified and analyzed in the sweet cherry genome. We systematically studied the gene structures and expression patterns of *PavJAZ* members. Furthermore, *PavJAZ8* was found to be responsive to MeJA. Transient overexpression of *PavJAZ8* in sweet cherry fruits broadly regulated the expression of genes involved in aroma volatile biosynthesis, suggesting its potential role in aroma trait development. Functional analysis revealed that *PavJAZ8* exerts dual effects on fruit aroma traits, demonstrating the complex and multifaceted role of *PavJAZ8* in regulating fruit aroma profiles. Protein interaction assays further confirmed that *PavJAZ8* physically interacts with *PavMYC2*. Our findings establish *PavJAZ8* as a key regulator of fruit quality and enhance our understanding of JA signaling in fruit ripening and trait development.

## Figures and Tables

**Table 1:** Basic physicochemical characteristics of *PavJAZs*.

Gene Name	Gene ID	CDS (bp)	Protein (aa)	Molecular Weight (Kd)	Isoelectric Point	GRAVY	Instability Index
*PavJAZ1*	FUN_000255-T1	1002	333	36.78	9.19	−0.853	46.60
*PavJAZ2*	FUN_003441-T1	801	266	29.23	9.79	−0.357	57.83
*PavJAZ3*	FUN_004843-T1	1134	377	40.50	8.61	−0.433	43.38
*PavJAZ4*	FUN_007360-T1	1047	348	40.24	9.80	−0.636	55.00
*PavJAZ5*	FUN_013768-T1	801	266	28.83	9.15	−0.391	50.91
*PavJAZ6*	FUN_032462-T1	363	124	14.66	9.60	−0.959	103.28
*PavJAZ7*	FUN_026609-T1	1125	374	39.32	9.40	−0.222	53.13
*PavJAZ8*	FUN_039026-T1	651	216	23.21	6.18	−0.667	66.52
*PavJAZ9*	FUN_039096-T1	837	278	30.09	9.21	−0.564	50.10

**Table 2:** Calculation of *Ka*/*Ks* of *PavJAZs*.

Gene Pair	*Ka*	*Ks*	*Ka*/*Ks*	Effective Len	Average S Sites	Average N Sites
*PavJAZ5/PavJAZ9*	0.5468	2.0797	0.2629	654	143.1667	510.8333

## Data Availability

All data generated or analyzed during this study are included in this published article.

## Supplementary Materials

The following supporting information can be downloaded at: https://www.mdpi.com/article/10.3390/biom15121721/s1, Table S1: Name and accession number of the genes involved in this study; Table S2. Primers used for real-time quantitative polymerase chain reaction; Table S3. Subcellular localization prediction of *PavJAZ* proteins. Figure S1: Analysis of *PavJAZ8* expression in response to ABA, SA, and GA_3_.
