# Supplementary material for: Investigation of the Jasmonate ZIM-Domain Family Reveals PavJAZ8 Regulates Fruit Aroma Traits in Sweet Cherry (Prunus avium L.)
Source: Biomolecules. 2025 Dec 11;15(12):1721. doi: 10.3390/biom15121721 (PMC12731118; doi:10.3390/biom15121721)

Original unedited, uncropped images for the pull-down assay

Anti-GST:

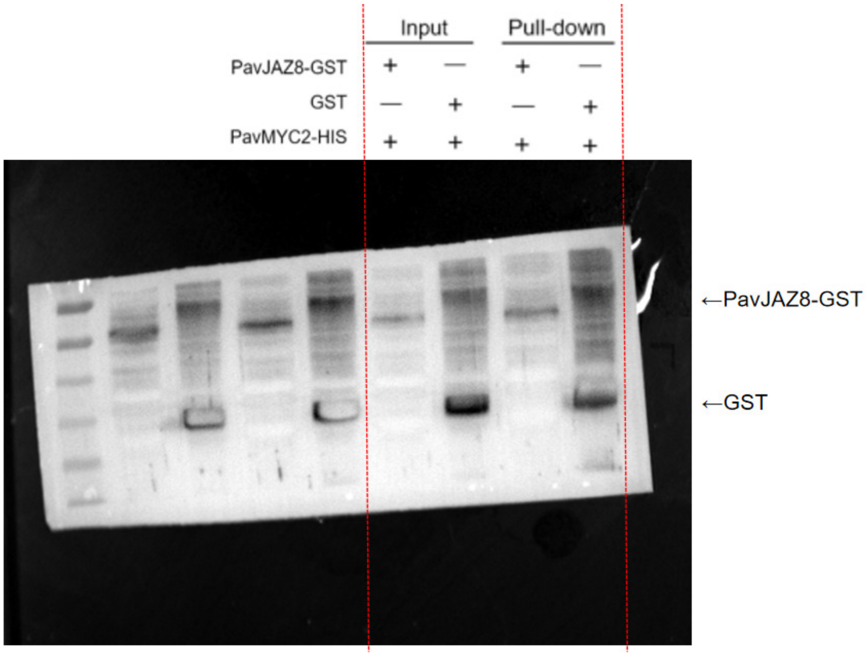

Original image:

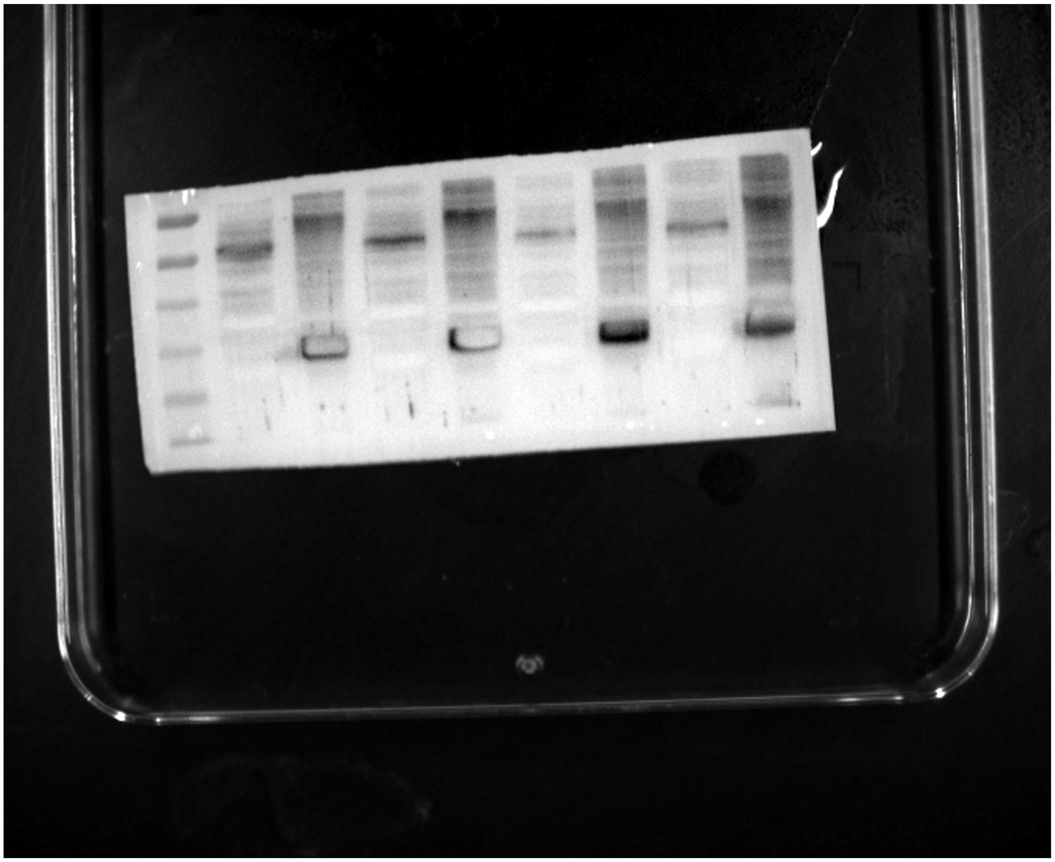

Anti-HIS：

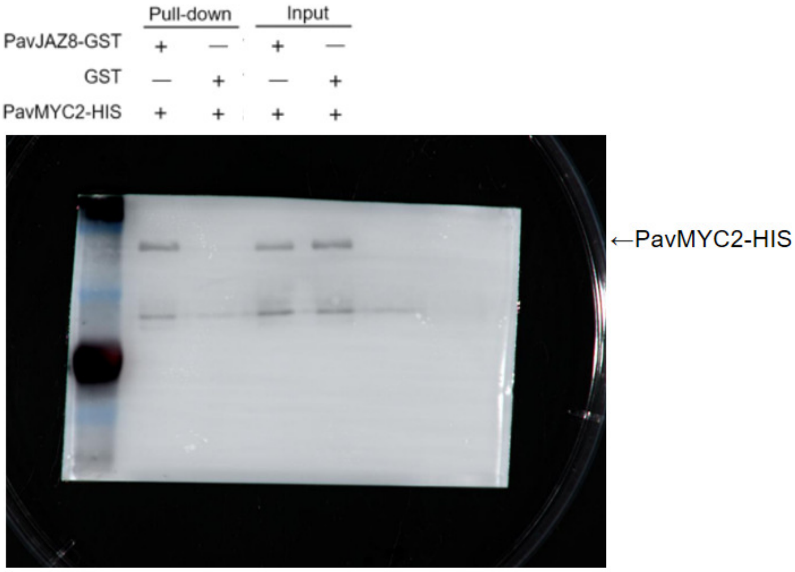

Original image：

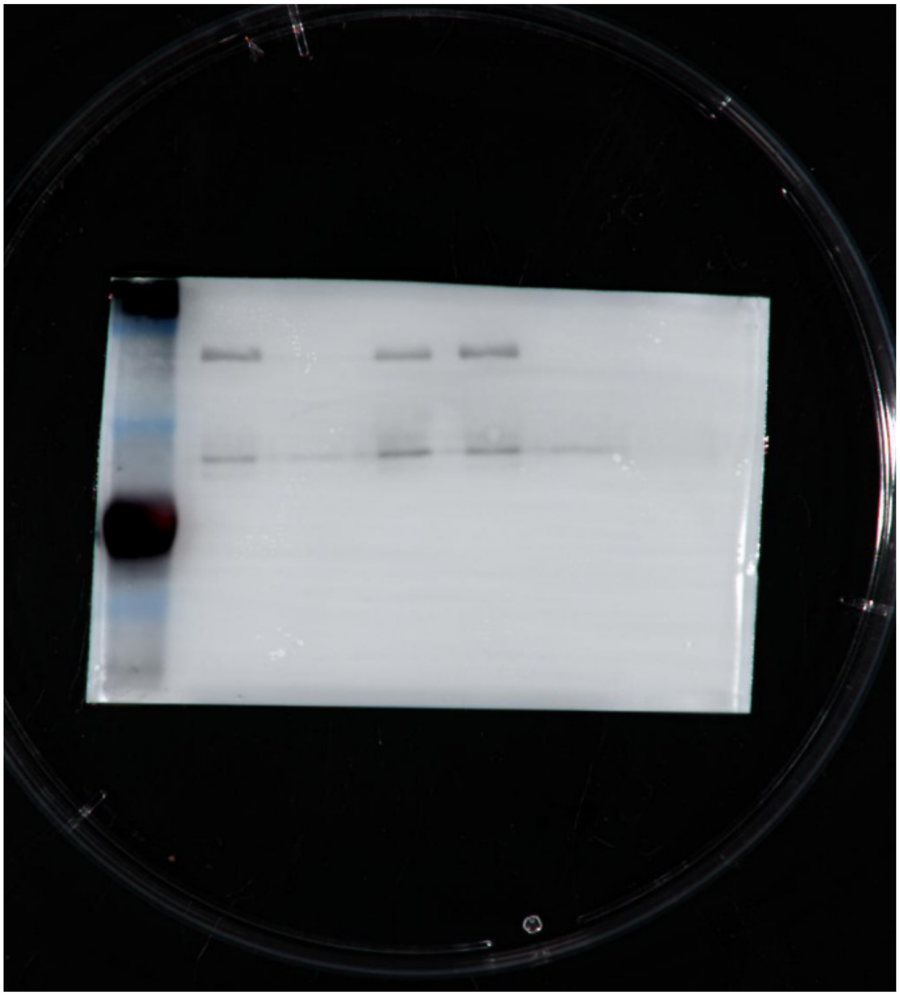

Supplement: Supplementary file 1 [file biomolecules-15-01721-s001.zip › Original images of Figure 7C.pdf]
