# Supplementary material for: Investigation of the Jasmonate ZIM-Domain Family Reveals PavJAZ8 Regulates Fruit Aroma Traits in Sweet Cherry (Prunus avium L.)
Source: Biomolecules. 2025 Dec 11;15(12):1721. doi: 10.3390/biom15121721 (PMC12731118; doi:10.3390/biom15121721)
Supplement: Supplementary file 1 [file biomolecules-15-01721-s001.zip › Table S1-S3; Figure S1.pdf]

Table S1. Name and accession number of the genes involved in this study.

Gene Name	Accession Number
PavJAZ1	FUN_000255-T1
PavJAZ2	FUN_003441-T1
PavJAZ3	FUN_004843-T1
PavJAZ4	FUN_007360-T1
PavJAZ5	FUN_013768-T1
PavJAZ6	FUN_032462-T1
PavJAZ7	FUN_026609-T1
PavJAZ8	FUN_039026-T1
PavJAZ9	FUN_039096-T1
PavMYC2	FUN_024373-T1
PavACTIN	FUN_020521-T1
PavLOX2	FUN_008062-T1
PavLOX3	FUN_013783-T1
PavLOX6	FUN_003619-T1
PavHPL1	FUN_016697-T1
PavADH1.1	FUN_027523-T1
PavADH1.2	FUN_027513-T1
PavADH1.3	FUN_027512-T1
PavADH7	FUN_007235-T1
AtJAZ1	AT1G19180
AtJAZ2	AT1G74950
AtJAZ3	AT3G17860
AtJAZ4	AT1G48500
AtJAZ5	AT1G17380
AtJAZ6	AT1G72450
AtJAZ7	AT2G34600
AtJAZ8	AT1G30135
AtJAZ9	AT1G70700
AtJAZ10	AT5G13220
AtJAZ11	AT3G43440
AtJAZ12	AT5G20900
AtJAZ13	AT3G22275
FvJAZ12	FvH4_1g09690

Gene sequence data in this article could be found by accession numbers provided from the Genome Database for Rosaceae and the TAIR database.

Table S2. Primers used for real-time quantitative polymerase chain reaction.

Primer Name	Forward (5'-3')	Reverse (5'-3')
PavJAZ1	TACTGCGGCAAGGTGAATGT	CCATCTTTGTCTCCGGCAGT
PavJAZ2	TAGACATGCCAGCAACAGGG	ATCGTCAGAGGCGTTGTTGT
PavJAZ3	CTGTTCCTGTGAGCAACCCT	GGTGCTCCCTGAAACTGGAA
PavJAZ4	ATCGTCAGAGGCGTTGTTGT	CTCTCGTCTGGGAACTTCGG
PavJAZ5	CCCAGAAGCAGTTTTGGCAG	TTCCCTTGGTAGCCAAACCC
PavJAZ6	ATGGAGTGATGTGCGTTCGT	TAGACATGCCAGCAACAGGG
PavJAZ7	GTTCAGGGATGCAGTGGTCA	ACCAGAGAATGGCTTCTGGC
PavJAZ8	TGCAAAACTCTGCTCCTGGT	GTGTTGCGGGTGAAGTAGGA
PavJAZ9	GCCATCGAGCTTCACTCAGA	CTGTCCCGTTGGATTGTTGC

Table S3. Subcellular localization prediction of PavJAZ proteins.

Gene Name	Cell-PLoc 2.0 Predicted Location(s)	WoLF PSORT Predicted Location(s)
PavJAZ1	Nucleus.	nucl: 12, cyto: 2
PavJAZ2	Nucleus.	chlo: 9, nucl: 5
PavJAZ3	Nucleus.	nucl: 13, cyto: 1
PavJAZ4	Nucleus.	nucl: 8.5, cyto_nucl: 5.5, cysk: 2, cyto: 1.5, chlo: 1, golg: 1
PavJAZ5	Nucleus.	chlo: 8, extr: 3, nucl: 2, mito: 1
PavJAZ6	Nucleus.	nucl: 12, plas: 1, extr: 1
PavJAZ7	Nucleus.	nucl: 11, cyto: 3
PavJAZ8	Nucleus.	nucl: 7, chlo: 5, mito: 1, plas: 1
PavJAZ9	Nucleus.	nucl: 10, nucl_plas: 6.5, chlo: 2, mito: 1

The subcellular localization of the PavJAZs was predicted by the online web-servers tool Cell-PLoc 2.0 (http://www.csbio.sjtu.edu.cn/bioinf/Cell-PLoc-2/) and WoLF PSORT (https://wolfpsort.hgc.jp/).

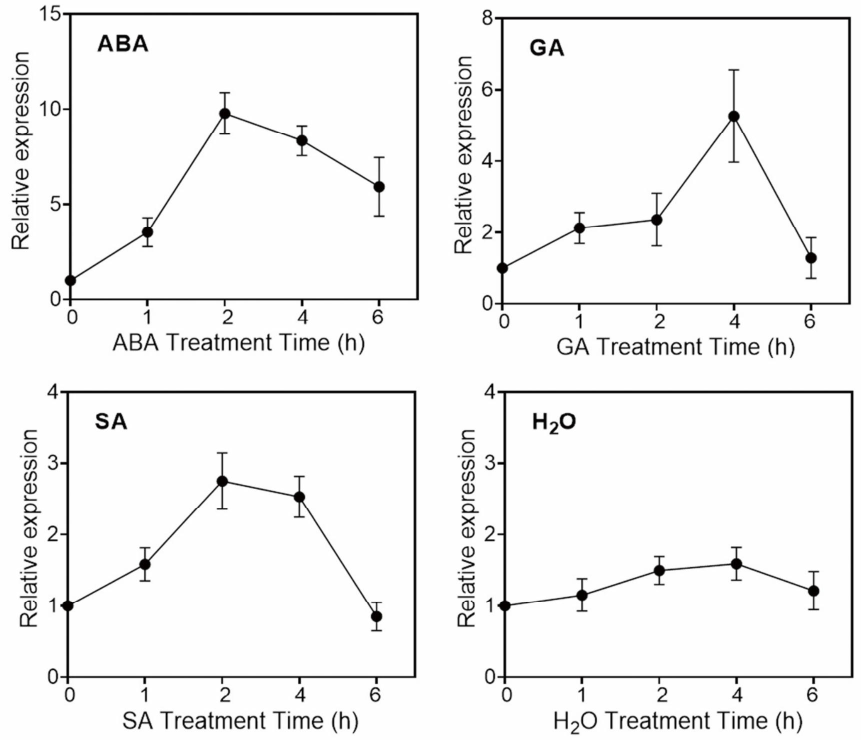

Figure S1. Analysis of *PavJAZ8* expression in response to ABA, SA, and GA_3. Analysis of *PavJAZ8* expression in response to ABA, SA, and GA_3 treatments by RT-qPCR, with H_2O as control. Values represent the mean ± SD from three independent biological replicates.
